# Recent Progress in the Treatment of Mental Diseases

**Published:** 1879-12

**Authors:** Theodore W. Fisher

**Affiliations:** Harv.


### RECENT PROGRESS IN THE TREATMENT OF MENTAL DISEASES.

By THEODORE W. FISHER, M.D., Harv.

*The Moral Nature of the Great Sympathetic.*—This is a paper read by Dr. R. M. Bucke, of London, Ontario, at the Association of Superintendents' meeting at Washington, last year, and is supplementary to another one read the year before at St. Louis, both of which are, we believe, parts of an essay by the same author, published last June. The mind, Dr. Bucke states, is made up of simple moral states, simple concepts, and of the infinite number of compounds formed from them. These compounds are of three kinds: (1) compounds of simple moral states, (2) compounds of concepts, (3) compounds of moral states and concepts. The elementary moral states are four, namely, faith, love, fear and hate. In children faith, or confidence, and love seem to exist for a time as one primative function. It is probable, also, that in animals, if not in children, the negative moral states of fear and hate are at first united. These simple states may be compounded with each other and with numerous complex concepts, so as to produce a great variety of feelings, passions and emotions. Opposite moral states cannot of course be compounded, as love with hate. As compared with concepts the moral states are few and simple, requiring a proportionately small organic apparatus. The brain, by its complexity, is fitted for motor, sensory and intellectual functions. The moral states have, however, a wide range of intensity. They are far more continuous also, emotional conditions lasting for hours and days sometimes, while ideas are exceedingly transient. The parallel between the known functions of the brain, sensation and motion, and those of the great sympathetic, nutrition and secretion, with the two sections of mind, intellect and feeling, is, the author thinks, very striking. The functions of the great sympathetic are continuous, have a wide range of intensity, and are intimately associated with emotional states.

Dr. Bucke argues from his own investigations, as well as from other sources, that there is an intimate relation between great vitality depending on the sympathetic and high morality. Persons of the highest moral nature live longest. He instances the Jews, men of distinction, women and married persons. He finds the average age of 13,539 persons mentioned in a cyclopædia of biography to be sixty-three and a half years, which is ten years greater than the average of Englishmen who have become established in a business or profession. Of 11,093 men of note who had passed the age fifty, one in thirty-two lived to be ninety, while by the English life-table one in forty-one only live to that age. Women live longer than men by from two to four years. The moral nature in women is more developed than in man, and the intellect less so. The great sympathetic is also larger, has more organs to supply, and the brain smaller, the average weight being forty-four ounces against forty-nine and a half ounces in man. The increased longevity in these four classes being associated with a higher morality, the hypothesis is warranted that the moral nature depends upon, or is a function of, the great sympathetic. This argument, it must be remembered, is only supplementary to a fuller treatment of the subject in the essay before mentioned. The moral nature, its limits, physical nature and development are there considered, with "the inference to be drawn from its development as to the essential fact of the universe."

*Psychometry.*—Francis Galton, F.R.S., reports a series of subjective experiments, intended to show the rate of the association of ideas, their character, date of formation, tendency to recurrence, and relative precedence. He finds that by patient effort a "whole strata of mental operations that have lapsed out of consciousness admit of being dragged into light, recorded and treated statistically, and that the obscurity which attends the initial steps of our thoughts can be pierced and dissipated." By the use of written words, disclosed to sight singly, chronometer in hand, he obtained the data of his observations. He noted carefully the time which elapsed before an associated idea occurred, and also the idea itself against its associated word, using each word four times over. He found that five hundred and five ideas required six hundred and sixty seconds, which is at the rate of fifty ideas per minute, or three thousand per hour. This would be slow if the mind were allowed to persue an uninterrupted train of ideas, as in reverie. The same ideas arose four times in twenty-three per cent. of cases, three times in twenty-one per cent., twice in twenty-three per cent. and once in thirty-three per cent. He believes that unless the mind often repeats an idea it loses it altogether. Recollections of early life he finds imperfect, though firmly fixed from frequent repetition. In old age the mind becomes less flexible, because it cannot help running in the well-defined tracks of familiar ideas. The multifarious work done by the mind in a state of semi-consciousness is startling, and seems to indicate a still deeper strata of ideas sunk below the level of consciousness. Though our ideas are marvelous for number and nimbleness, they are far from infinite in variety, and our working stock of them is quite limited.

*General Paralysis.*—Dr. Bucknill, in discussing the pathology of general paralysis, claims to have pointed out its origin more than twenty years ago by showing the gradual failure of reflex action in this disease. He thinks the cerebral and spinal conditions which usually co-exist are in a measure independent of each other, and that the chronic sub-inflammatory state of the meninges is not the fundamental morbid process. This he considers to be some undiscovered chemical or nutritive change affecting the whole nervous system. He says that Westphal has failed to give due credit to his observations. In the article mentioned Westphal distinguishes two forms—the tabic, in which the motor symptoms precede, due to gray degeneration of the posterior columns of the cord, and the paretic, in which the mental phenomena, usually dementia and optimism, are well advanced before motor symptoms supervene. We recently examined the brain in a case of general paralysis of the tabic form. The mental symptoms had been characteristic, although late in occurring, and the naked-eye appearances, such as opacity of the arachnoid, effusion, shrinking of the convultions and spots of decortication, were distinct. In its earliest stages the case had been diagnosticated by a competent neurologist as locomotor ataxia.

*The Chemical Pathology of the Brain* is the subject of a paper by Dr. Addison. He experimented on the brains of twelve insane persons, and found that different parts of the same brain presented greater differences in their quantities of water and fat than in the sane; that the gray substance is poorer in fat than the white; that the matters soluble in ether stand in inverse relation to the quantity of water; that there is slightly more water and less fat than in sane brains; that in two cases of idiocy, one of dementia and one of chronic melancholia, the fat was less in quantity than in the brain of a new-born child; and that the quantity of phosphorus showed no parallel connection with the degree of intelligence.

*New Type of Insanity.*—The members of the German Verein of Psychiatrie adopted last year a resolution, proposed by Dr. Meynert, recognizing, besides mania and melancholia, a third form of mental disease, or primary insanity, described by Twigges as *Wahnsinn.* It is usually due to hereditary neurotic tendency. The patient is soft, morbidly sensitive, conceited, theatrical, mystical, or hypochondriacal; always thinking of the conduct of strangers as regards him, often imagining that he is persecuted or ill-used. Outbreaks of excitement are rare, or illusions or hallucinations only occasionally present. The self-accusation of melancholia is wanting, as well as the active aggressiveness of mania. Cases of this type were reported to be as numerous in the Vienna asylum as those of mania and melancholia together.

*Periodical Melancholia.*—William B. Neftel, M.D., in a paper read before the New York Medical Library Association, treats of this not infrequent form of insanity. His remarks are based upon a case in which the disease had occurred almost annually for a period of twenty years, without developing any other form of insanity or tending to dementia. The recovery from each attack was complete, and often sudden. The attack lasted from four to eleven months, and the interval was about five months.

Loss of flesh was an invariable accompaniment, followed by increase in weight on recovery. The attacks began with subacute anæmia, soon becoming general. The skin and mucous membranes were pale; the pulse was about 55, small and feeble; the veins dilated and distended with blood. There was a morbid alteration of nutrition, as shown by boils, eruptions, itching of the skin, and splitting of the hairs of the beard, with a diminution of all the secretions.

Dr. Neftel remarks that Guislain was the first to call attention to the fact that "the great majority of all mental diseases being with a melancholic stage, while Griesinger treated this psychical affection in close relation to nervous diseases, and Krafft-Ebing pointed out the analogies of melancholia and neuralgia. Indeed, melancholia can be considered as a psychical pain, a neurosis, a psychical neuralgia of the sensory centres in the cortical substance of the brain." There is a hyperæsthesia of these centres, so slight external irritation will produce extreme mental diseases. From the study of periodical melancholia Dr. Neftel concludes that its direct cause is anæmia of the brain or of some part of it, induced by vaso-motor spasm. Other signs are observed in the contracted pulse, pallor and flushing, cold extremities, and præcordial distress. The spasm is not constant, but probably recurs so frequently as to impair nutrition.

The treatment should be directed against this neuropathic condition. At the outset of the attack a few days' rest in bed are desirable, with remedies tending to the relief of spasm, such as tepid baths and opiates, which make life endurable and palliate the præcordial distress. Inhalations of ether, chloroform, nitrite of amyle, chloral, etc., may be tried. In the case in question, having exhausted all the known electro-therapeutic methods, the patient was sent in the country for the summer. On his return in the fall, without any change in his condition, he recovered after six days' treatment by a new plan, namely the production by the polar method of anelectrotonus in the cervical sympathetic. This was followed by refreshing sleep, with a speedy disappearance of all the physical and mental derangements.

We are tempted to comment on this suggestive paper, to speak of occasional sudden recoveries from melancholia, and of cure, permanent and perfect, after long attacks, of five years' duration, even in persons of advanced age. Such instances, as well as the almost certain recovery in periodic cases, should be used as means of encouragement in the treatment of mental depression. We have found message of great benefit in a periodical case; also mono-bromated camphor. One patient got well suddenly from a third annual attack while camping out on the plains of Texas, and has remained so for three years. The cloud lifted, the gloom disappeared, the leaden cap was raised in a single night. Now and then the patient hears a snap, or feels something give way in his head, and is immediately relieved.—*Boston Medical and Surgical Journal.*

[*To be concluded.*]

